# Identification and Characterization of *Fusarium fujikuroi* Pathotypes Responsible for an Emerging Bakanae Disease of Rice in India

**DOI:** 10.3390/plants12061303

**Published:** 2023-03-14

**Authors:** Prashantha S. Tadasanahaller, Bishnu Maya Bashyal, Jagdish Yadav, Gopala Krishnan Subbaiyan, Ranjith K. Ellur, Rashmi Aggarwal

**Affiliations:** 1 Fungal Molecular Biology Laboratory, Division of Plant Pathology, ICAR-Indian Agricultural Research Institute, New Delhi 110012, India; 2 Division of Genetics, ICAR-Indian Agricultural Research Institute, New Delhi 110012, India

**Keywords:** Basmati, differential, isolates, PB1509, susceptible

## Abstract

The bakanae disease of rice, or foolish seedling disease, is a well-known pathogen infecting rice hosts. Several studies have characterized *Fusarium fujikuroi* isolates collected from distant geographical regions and within similar geographical areas for secondary metabolite production, population structure, and diversity analysis, but none have attempted to characterize the isolates for virulence in a differential set of rice genotypes. Based on the disease response, a set of five rice genotypes with differing resistance levels were selected as a differential set for further characterization of the pathogen. Ninety-seven *Fusarium fujikuroi* isolates collected from different rice-growing areas of the country during the years 2011 to 2020 were characterized and evaluated for bakanae disease. Rice genotypes PB1509 and C101A51 were found to be highly susceptible and highly resistant, respectively. Further, based on the disease response, the isolates were grouped into 15 pathotypes. Pathotype 1, with the maximum isolates (19), was observed to be most prevalent, followed by pathotypes 2 and 3. Pathotype 8 was classified as highly virulent, as all the genotypes were susceptible, except for C101A51. When we compared the pathotype distribution in different states, pathotypes 11 and 15 were found to have originated from the state of Punjab. A positive correlation could be established between six pathotype groups and the gene expression of virulence-related genes such as acetylxylan (*FFAC*), exopolygalacturanase (*FFEX*), and pisatin demethylase (*FFPD*). The present study provides the distribution profiles of different pathotypes in Basmati-growing states of India, which will be further helpful for the deployment of breeding strategies and bakanae disease management.

## 1. Introduction

Fungal diseases in rice are the biotic factor with the most relevance in crop yield production. One of the most important and unique diseases in rice crops is bakanae, or foolish seedling disease, caused by *Fusarium fujikuroi* [1]. The first description of the disease was given by Hori [2]. The key identification feature of the disease is the elongation of seedlings, or “bakanae” (foolish seedlings). The disease is distributed around the world, which includes major rice-growing countries such as China, India, Indonesia, Malaysia, the Philippines, Thailand, Japan, America, and African countries [3,4,5,6]. In India, the disease is prevalent in Punjab, West Bengal, Uttar Pradesh, Assam, and, more particularly, in the Basmati-growing regions of Haryana, Punjab, and Uttar Pradesh [6,7,8,9].

*Fusarium fujikuroi* infection in rice plants produces various symptoms, such as elongated pale-yellow seedlings, drying, rotting, seedling blight, rotting of roots, stunting of plants, and discoloration of grains [10,11,12]. The whitish mycelium of the fungus can be seen on the surface of the lower stem parts of the rice plants. The primary inoculum for the bakanae disease is infected seeds [13,14], but it can also survive in soil and plant debris. It is a weak soil inhabitant but the survival rate decreases with time [15]. The favorable temperature required for the development of disease is 35 °C, with high relative humidity. The disease can cause up to 70% yield losses under favorable environmental conditions [16,17]. In India, bakanae is more seriously affects Basmati varieties of rice and yield losses of 15–25% have been reported [8,18,19,20,21]. Understanding the pathogen infection mechanism, the diversity within the population, and their virulence will be useful in the development of suitable management practices/technologies. *Fusarium fujikuroi* (erstwhile referred to as *Fusarium moniliforme*) isolates were classified into five virulence groups, five GA3 (gibberellic acid production) groups, and 10 VCG (vegetative compatibility) groups [22,23]. The study also revealed that GA3 production was positively correlated with the virulence of the isolates [22,23]. Similar studies on the pathogenic behavior of isolates were conducted by several other researchers [24,25]. *Gibberella fujikuroi* species complex (GFSC) was isolated from rice seed samples of Asian countries, and studies found that *Fusarium fujikuroi* was the predominant species associated with rice seed samples, along with *F. concentricum*, *F. proliferatum*, and *F. verticilliodes* [26]. In Basmati-growing states of India, bakanae disease was surveyed and 126 isolates were recovered from the infected plants. The Basmati varieties were found susceptible to the disease. The isolates were characterized for their morphological features and pathogenicity, grouped into moderately virulent (37.3%), virulent (34.1%), and highly virulent (28.6%) isolates based on their disease response in rice variety Pusa Basmati 1121 (PB1121) [6]. Pathogen genome information and molecular characterization will help to unravel the mechanism of infection and pathogenicity-related genes involved in causing the disease. Genome-wide analysis of *Fusarium fujikuroi* indicated novel genes and secondary metabolites involved in bakanae disease in rice [27]. Whole-genome sequencing of *Fusarium fujikuroi* isolate F250 revealed that secretory proteins and cell-wall-degrading enzymes play an important role in causing bakanae disease in rice [28]. Similarly, a putative gene involved in causing bakanae disease has been identified through the comparative sequencing of non-virulent and virulent *Fusarium fujikuroi* isolates [29]. The use of resistant varieties is the most sustainable method of plant disease management. Currently, high-yielding resistant varieties are not available against bakanae disease. Several attempts were made to identify resistant sources against bakanae disease. Fiyaz and coworkers [30] screened rice genotypes for bakanae disease, and the genotypes C101A51 and Pusa 1342 were identified as highly resistant, while PB1121, Pusa 1568, and Rasi were identified as highly susceptible. Pathotype identification using rice genotypes with known resistance/susceptibility to bakanae disease were used by researchers to characterize *Fusarium fujikuroi* isolates, but the pathotype itself has not been characterized. In the present study, *Fusarium fujikuroi* isolates were evaluated for disease incidence and they were classified into pathotype groups based on the disease response patterns of five rice genotypes differing in their resistance. Further, pathotypes were evaluated for pathogenicity-related genes to establish the pathotypes and virulence.

## 2. Results

### 2.1. Screening and Selection of Differential Rice Genotypes

All rice genotypes have varied degrees of resistance to *Fusarium fujikuroi* infection. Genotypes such as Pusa Basmati 1509 (PB1509), PB6, K-14, BPT5204, and PS-5 were found to be susceptible (S), PB1121 was highly susceptible (HS), PB1 and ANP 115-3-3-3-3 were moderately susceptible (MS), Kanak-Jeer and IRG52 were moderately resistant (MR), C4-63G was resistant (R), and C101A51 was highly resistant (HR) (Table 1). The effect of genotype in the ANOVA test was found to be significant (Supplementary Table S1). Among these, five genotypes—namely PB1, PB6, PB1509, IRG52, and C101A51—were selected for further evaluation and the identification of pathotypes. The criteria for selection were based on their patterns of disease reaction and corresponding disease incidence values with a multiple comparison test (Table 1; Supplementary Table S2). The highest disease incidence values were used for susceptible/highly susceptible classes and the lowest values in the case of resistance/moderate resistance/moderately susceptible classes. PB1121 was used as a susceptible control.

### 2.2. Evaluation of Fusarium fujikuroi Isolates and Disease Pattern in a Set of Rice Genotypes

**Figure 1:**
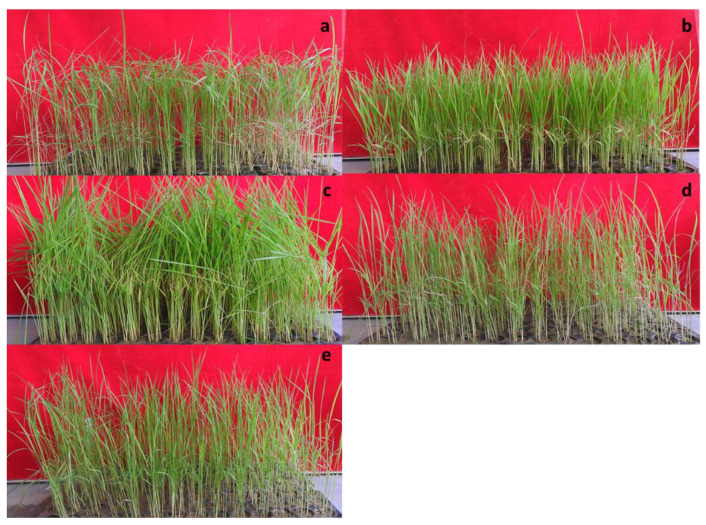
**Rice genotypes inoculated with *Fusarium fujikuroi* isolates and appearance of typical bakanae symptoms**. (**a**) PB1509 (susceptible), (**b**) C101A51 (highly resistant), (**c**) IRG52, (**d**) PB1, (**e**) PB6.

**Figure 2:**
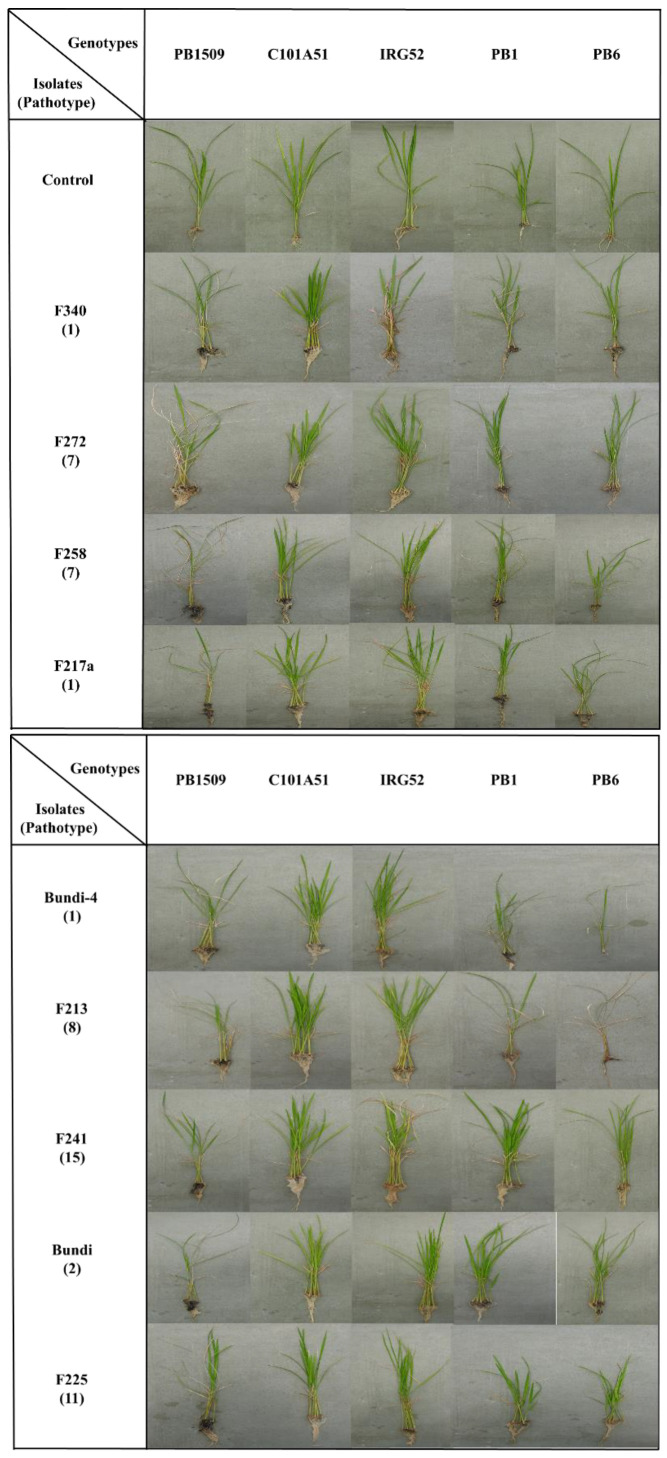
**Representative isolates causing bakanae disease of rice in genotypes differing in their resistance and their corresponding pathotype group**. Blackening of roots and drying of the plants can be observed in PB1509, PB1, and PB6, but C101A51 and IRG52 possess intact roots and green leaves. Pathotypes were classified based on the disease response of five rice genotypes differing in their resistance.

**Figure 3:**
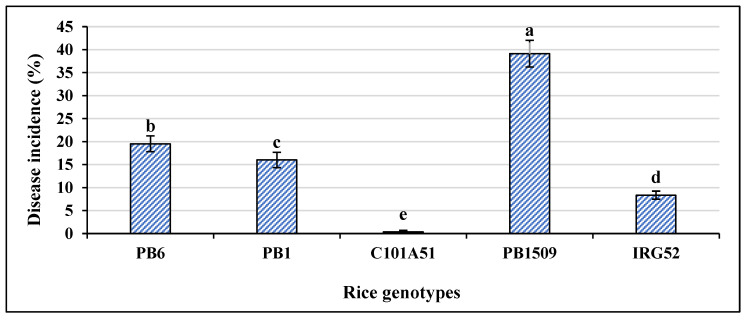
**Disease incidence (%) in rice genotypes differing in their resistance.** The disease was highest in susceptible control, followed by PB1509, PB6, PB1, and IRG52, and was the lowest in C101A51. The disease incidence is the mean of all 97 isolates in five rice genotypes. Alphabetic letters denote significant differences between the genotypes. Error bars represent standard error.

**Figure 4:**
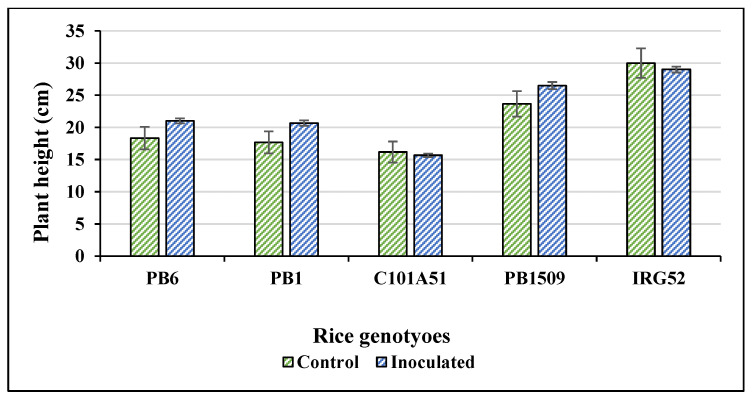
**Elongation of the rice genotypes due to infection by *Fusarium fujikuroi*.** Significant elongation in plant height was observed in Basmati rice varieties (PB6, PB1, and PB1509) as compared non-Basmati genotypes (C101A51 and IRG52—no significant elongation/stunting was observed). Mean plant heights of all treatments (all combinations) were taken to plot the graph (in each replication, 3 plants were recorded). Error bars represent standard error.

**Figure 5:**
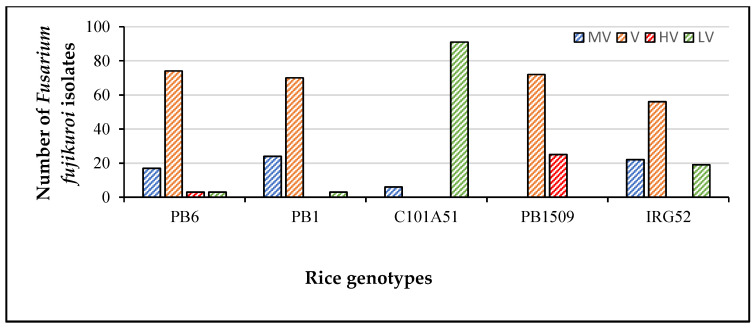
**Virulence pattern of *Fusarium fujikuroi* isolates in rice genotypes differing in their resistance.** Isolates were classified into moderately virulent (MV), virulent (V), highly virulent (HV), and less virulent (LV) based on degree of pathogenicity.

A total of 485 combinations were made with 97 isolates and a set of five rice genotypes, each having three replications. PB1121 was used as a susceptible control and inoculated with highly virulent isolate F250. The disease was first noticed in PB1121 as drying of the young leaves 13–14 days after sowing. The rice genotypes were inoculated with *Fusarium fujikuroi* isolates along with controls (Supplementary Figure S1). *Fusarium fujikuroi* infection produced differences in macroscopic symptoms, such as green intact leaves in C101A51 and IRG52 as compared to other genotypes (Figure 1). The isolate and genotype interaction produced a HS reaction to HR reaction in rice genotypes differing in their resistance. The symptoms such as drying, drooping, elongation, yellowing of leaves, and blackening of roots, particularly in susceptible variety PB1509, were observed and the disease reactions of rice genotypes for some selected isolates were also recorded (Figure 2). The disease incidence (%) data of all the isolates are given in Table 2. The maximum disease incidence was observed in PB1509, with the maximum average disease incidence of 39.13%, and the lowest average disease incidence of 0.35% was found in C101A51. Thus, the interaction between isolate and genotype can be classified as resistant (C101A51) or susceptible (PB1509). The disease incidence was highest in PB1509, followed by PB6, PB1, IRG52, and C101A51 (Figure 3). Varieties such as PB1509, PB6, and PB1 produced significant elongation as compared to controls, C101A51, and IRG52 (Figure 4, Supplementary Table S3). Elongation in C101A51 and IRG52 was not significant as compared to the control, indicating the resistance reactions of these genotypes (Figure 4). All isolates were able to infect the genotype PB1509. The isolates F222, F224, F226, F242, F242a, and F256 caused >90% infection in PB1509, whereas the same isolates did not cause infection in C101A51. Other genotypes, such as PB6, PB1, and IRG52, can be classified as moderately susceptible (MS) based on their mean disease incidence values (Table 2). Isolates F213, F220, F226, F233, F239, and F240 produced >30% mean disease incidence across the five rice genotypes, and the isolates F214, F255a, F297, and F344 caused <5% mean disease incidence (Table 2). The disease incidence (%) was classified into different levels of disease reaction pattern (Supplementary Table S4) according to the disease rating scale. *Fusarium fujikuroi* isolates were grouped into moderately virulent (MV), virulent (V), highly virulent (HV), and less virulent (LV) based on the degree of pathogenicity in rice genotypes differing in their resistance (Figure 5).

### 2.3. Multiple Comparisons among the Fusarium fujikuroi Isolates

The two-way ANOVA test indicated significant differences between isolates (Supplementary Table S5). Therefore, we compared the isolates using Tukey’s HSD test. Among the 97 isolates, F213 had a significant positive effect on the disease incidence, followed by F239 (Supplementary Table S6). The isolates F220 and F226 can be classified as one group and F233 as another group. Further, the isolates F287, F297, F214, F344, and F255a had no significant positive effect on the disease as compared to other isolates. All other remaining isolates can be classified as one group with a significant positive effect in the development of bakanae disease.

### 2.4. Pathotype Classification of Fusarium fujikuroi Isolates

A total of 15 pathotype groups were identified based on the responses of the rice genotypes to the *Fusarium fujikuroi* isolates (Table 3). The pathotype group 1 consists of 19 isolates that showed susceptible reactions in all rice genotypes except C101A51, followed by pathotype 2 and pathotype 3, which include 13 and 9 isolates, respectively. The pathotype group 1 is more prevalent as compared to other groups. The pathotype group 12 showed the maximum variability in virulence pattern, with HS, S, HR, HS, and S reactions in PB6, PB1, C101A51, PB1509, and IRG52, respectively. The pathotype group 4 showed an S reaction in PB6, PB1, and IRG52, while a HS reaction was observed in PB1509 and HR in C101A51. The responses of rice genotypes to some representative *Fusarium fujikuroi* isolates and their corresponding pathotype groups are given in Figure 2. The isolates in pathotype group 8 showed a susceptible reaction in all rice genotypes except C101A51. This group can be classified as a highly virulent one and consists of six isolates, F213, F244, F250, F274, F277, and F343 (Table 3). Further, the isolate F213 was separated from other isolates due to its virulence (Supplementary Table S6). It is also interesting to observe the pattern of virulence in pathotypes 13 and 15, which produced a resistance reaction in all genotypes (in general) except PB1509, having a susceptibility reaction (Table 3).

### 2.5. Pathotype Distribution in Basmati-Rice Growing States of India

A correlation between pathotype groups of *Fusarium fujikuroi* isolates and the state of collection could not be established. Pathotype 1 consisted of isolates from all rice-growing states. However, isolates of pathotypes 11 and 15 were originated from Punjab. Pathotype 7 was also originated from Punjab except one isolate F309, which belong to Uttar Pradesh. Similarly, pathotype 8, which was found to be highly virulent, was distributed in Punjab and Haryana (Table 4). 

### 2.6. Expression Analysis of Virulence-Related Genes in Fusarium fujikuroi

**Figure 6:**
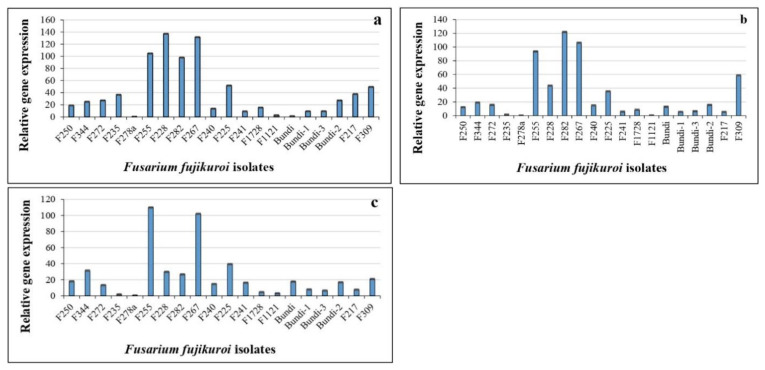
**Relative expression of virulence-related genes in selected *Fusarium fujikuroi* pathotypes.** (**a**) Acetylxylan (*FFAC*), (**b**) exoploygalacturanase (*FFEX*), and (**c**) pisatin demythalase (*FFPD*). The relative gene expression data were used for grouping of *Fusarium fujikuroi* isolates and pathotype correlation. Error bars represent standard error.

**Figure 7:**
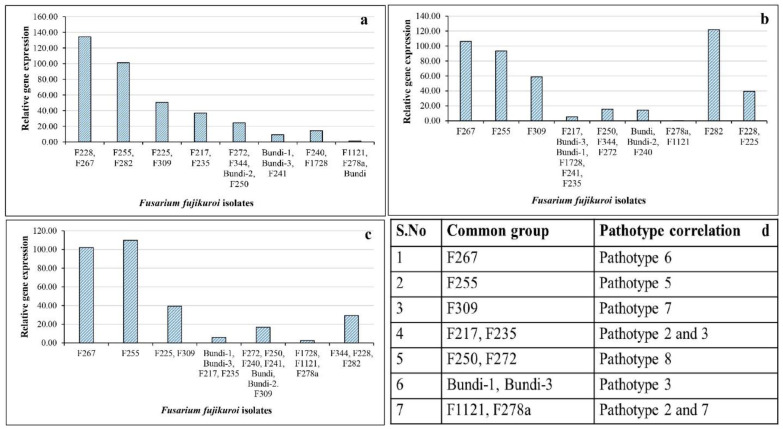
**Grouping of *Fusarium fujikuroi* isolates based on virulence-related gene expression and pathotype correlation**. (**a**–**d**) Grouping of isolates based on gene expression analysis: (**a**) acetylxylan (*FFAC*), (**b**) exoploygalacturanase (*FFEX*), (**c**) pisatin demythalase (*FFPD*), and (**d**) common groups were formed by comparing the isolates in all three genes and used for pathotype correlation. Pathotypes 2 and 3, and pathotypes 2 and 7, were considered as single groups. In each gene, isolates showing similar fold change values were classified into groups and mean fold change values were used to plot the graph. In total, six pathotype groups have positive correlations with virulence-related gene expression analysis.

To correlate pathotype groups and virulence-related gene expression, three genes were selected from a previous study [29]. Expression patterns of three virulence-related genes, viz., acetylxylan (*FFAC*), exopolygalacturanase (*FFEX*), and pisatin demethylase (*FFPD*), were recorded across representative isolates of pathotype groups. These isolates showed significant fold changes in their virulence-related gene expression (Supplementary Table S7). Isolates F250 (pathotype 8), F344 (pathotype 13), and F272 (pathotype 7) showed similar patterns of gene expression, with 15–30-fold upregulation for all three genes (Figure 6). The isolates F255 (pathotype 5) and F267 (pathotype 6) showed the most significant upregulation of all three genes as compared to other isolates. The isolate F225 (pathotype 11) showed a similar expression pattern, with the upregulation of all three target genes by 40–50 fold. Comparatively, Bundi isolates (representing pathotypes 1 to 4) originating from Rajasthan did not show much upregulation for all three genes (Figure 6). Apart from these, the expression levels of *FFAC*, *FFEX*, and *FFPD* were highest in the isolates F228 (pathotype 1), F282 (pathotype 5), and F255 (pathotype 5), respectively (Figure 6). Based on this information, isolates showing similar fold change expression were grouped and compared with pathotype grouping. A correlation between gene expression and pathotype grouping was observed for six pathotype groups (pathotypes 2, 3, 5, 6, 7, and 8). Common groups were formed based on gene expression data and the groups were compared with 15 pathotypes (Figure 7). A total of six groups had correlations with pathotype grouping. 

## 3. Discussion

Bakanae disease of rice, caused by *Fusarium fujikuroi*, is one of the emerging diseases in India and other rice-growing countries. We collected *Fusarium fujikuroi* isolates from major rice-growing states of the country to study the disease severity and diversity of the isolates, which could help to classify the isolates into specific groups or pathotypes. Initially, rice genotypes were screened for bakanae disease and a set of five rice genotypes were selected to study the disease incidence pattern, along with susceptible control PB1121. Some of these genotypes were identified in an evaluation of rice genotypes against bakanae disease [30]. The rice genotypes varied significantly in their disease reactions to the bakanae isolates. PB1509 was highly susceptible to the pathogen, whereas C101A51 did not show any symptoms, or very few plants were infected, indicating a highly resistant reaction. These two genotypes assisted in the classification of the bakanae disease incidence into two distinct groups, i.e., susceptible and resistant reactions. Therefore, these two genotypes can be used in any bakanae disease experimental studies. Bashyal and coworkers [6] collected *Fusarium fujikuroi* isolates from different rice-growing areas and classified them into three groups based on morphological characteristics such as mycelium color, macroconidia, microconidia, chlamydospores, and chains. Further, they classified the isolates into moderately virulent (37.3%), virulent (34.1%), and highly virulent (28.6%). In the present study, we observed that Basmati varieties were more susceptible to the disease as compared to non-Basmati varieties. To study the prevalence and occurrence of bakanae disease in Northern India, field surveys were conducted from 2006 to 2014. Disease incidence in Basmati varieties of rice was recorded and it was found that most of the Basmati varieties were susceptible [21]. Rice genotypes in this study varied significantly in their disease reaction. Fiyaz and coworkers [30] classified rice genotypes into HR (Athad Apunnu, C101A51, Chandana, IR 58025B, Panchami, PAU 201, Pusa 1342, and Varun Dhan), resistant (BPT 5204, Himju, Peeli badam, Suphala), MS (Pusa Basmati 1, Pusa Basmati 1509), and HS (Rasi and TKM 6, 31). PB1509 was classified into the MS group by Fiyaz and coworkers [30]. However, in the present study, Pusa Basmati 1509 (PB1509) was found to be highly susceptible to the bakanae disease. The *Fusarium fujikuroi* isolates in our study differed significantly in their ability to cause the disease on a set of rice genotypes differing in terms of their resistance, and the two-way ANOVA indicated a significant difference between isolates. Similar observations were made earlier, while studying the bakanae isolates for their virulence, GA3 production, and vegetative incompatibility. Further, isolates were grouped into five virulence groups, five GA3 groups, and 10 VCG groups. The study also revealed that GA3 production was positively correlated with the virulence of the isolates [22]. Around 172 isolates isolated from California rice and two from water grass were grouped into six unique AFLP haplotypes corresponding to six VCG groups. Among the six haplotype groups, two groups consisted of 94% of isolates [31]. Pathotype identification using differential sets with known resistance/susceptibility was used by researchers to characterize the isolates. Pathotype identification will be helpful to understand the variability of the pathogen within a population. The knowledge of the prevalence of a pathotype is necessary for the effective deployment of genes/QTLs in a breeding program for the development of resistant varieties. This will also help in deploying management methods to overcome a target pathotype. We have identified 15 pathotype groups based on the responses of the rice genotypes. Pathotype group 1 consists of 19 isolates that showed a susceptible reaction in all rice genotypes except C101A51, which is highly resistant. Similarly, pathotype group 8 produced susceptible reactions in all genotypes except C101A51. The isolate F250 is a member of group 8, which indicates that our data are in agreement with previous studies highlighting the F250 isolate as a highly virulent one [28]. On the contrary, pathotype group 15 produced a resistant reaction in PB6, PB1, IRG52, and HR in C101A51 and a susceptible reaction in PB1509. Comparative genomic studies revealed that at least two pathotypes exist in *Fusarium fujikuroi* correlating with secondary metabolite production. The elongation type will produce GA3 and the stunting type produces fumonisins [32]. However, the temperature factor has been reported to play a role in the development of the disease. With an increase in temperature, the disease is aggravated and isolates produce different symptoms, with minor modifications in their genomes [33]. In another study, 52 *Fusarium oxysporum* f.sp. *lentis* isolates were characterized for their virulence, and the authors identified seven pathotypes in *Fusarium oxysporum* f.sp. *lentis* [34]. 

In terms of virulence-related gene expression for the selected isolates, F250, F344, and F272 isolates have similar patterns of gene expression for all three genes, namely acetylxylan (*FFAC*), exopolygalacturanase (*FFEX*), and pisatin demethylase (*FFPD*). The upregulation of all three genes was observed in the isolates F255 and F267 as compared to other isolates. Carbohydrate esterases, belonging to a large group of carbohydrate-active enzymes, catalyze the removal of ester substituents from the glycan chains of polysaccharides. Acetylxylan esterases are a class of lingocellulosic enzymes involved in the degradation of the plant cell wall [35]. Other enzymes, such as exopolygalacturonase, also have similar mechanisms [36], which are reported to be upregulated during *F. oxysporum* infection in leguminoses [37]. The disruption of the gene pisatin demethylase results in the loss of virulence in *Nectria haematococca* [38], and it is also a virulence factor in both *F. oxysporum* and *F. solani* [39]. These genes were validated through qPCR and expression was found highest in virulent strains of *Fusarium fujikuroi* as compared to less virulent or non-virulent strains, which showed low to no expression [29]. Based on disease severity as well as virulence-related gene expression, six pathotype groups were identified. However, we could not determine a correlation among the expression patterns of the three virulence-related genes across all 15 pathotype groups, as the selected genes may be not sufficient to discriminate all the isolates. Further studies with a larger number of *Fusarium fujikuroi* isolates as well as virulence-related genes will help to clearly differentiate the isolates into pathotype groups.

## 4. Materials and Methods

### 4.1. Fungal Isolates and Rice Genotypes

*Fusarium fujikuroi* isolates collected during the years 2011–2020 were used in the study (Supplementary Table S8). Some of these isolates were originally collected and characterized by Bashyal and coworkers [6,40]. However, the culture collection has been enriched with a greater number of isolates (collected from 2016 to 2020). Promising rice genotypes (Supplementary Table S9) were used to characterize the *Fusarium fujikuroi* isolates. Plants were grown at the National Phytotron Facility, IARI, New Delhi.

### 4.2. Inoculation and Sowing Method

*Fusarium fujikuroi* isolates were maintained in potato dextrose agar (PDA) slants. The isolates were transferred to new PDA slants using the mycelium of the fungus. The PDA slants were incubated at 26 ± 2 °C for 6–7 days and used for spore suspension preparation. Autoclaved distilled water of approximately 5–6 mL was placed inside the PDA slants and the mycelium was scraped slowly with a sterile inoculation needle, mixed properly in the water to obtain a suitable spore concentration. The suspension was filtered with two layers of sterile muslin cloth and a spore concentration of 1 × 10^6^ per mL was used to inoculate the seeds [6]. The seeds (around 250 g of each genotype) were disinfected by dipping in 70% ethanol for 60 s and sequentially transferred into 1% sodium hypochlorite, three times with autoclaved distilled water, each for 60 s. Chaff and broken seeds were removed and true seeds were air-dried on sterile blotter paper. Fifty seeds were placed in a small 2 mL Eppendorf tube and 2 mL spore suspension was poured into the Eppendorf tubes. The inoculated seeds were incubated at 26 ± 2 °C for 48 h. Protrays containing 98 wells were filled with autoclaved field soil and watered before sowing. Infected seeds (25 seeds in each well) were sown via the dibbling method and three replications were maintained for each genotype and isolate. The growth conditions of the growth chamber were 30–35 °C temperature during the day and 16–18 °C during the night, with relative humidity of 80–85%. A hand sprayer was used to create a mist in the aerial environment of the growth chamber. Plants were watered by pouring water below the trays. For the control, the seeds were dipped in sterile distilled water and incubated similarly to the above conditions. The susceptible variety Pusa Basmati 1121 (PB1121) was used as a susceptible control for the disease.

### 4.3. Screening of Rice Genotypes

The rice genotypes used in the current study (Supplementary Table S9) were selected from 500 previously screened rice genotypes for bakanae disease. In preliminary screening, to identify a differential rice genotype against *Fusarium fujikuroi*, a set of 12 genotypes (Supplementary Table S9) were evaluated with 20 *Fusarium fujikuroi* isolates. Seeds were inoculated and sown as described above. The disease rating scale of 0 = HR, 1 = R, 3 = MR, 5 = MS, 7 = S, and 9 = HS (Table 5) was used to classify the disease reaction pattern according to the method described in the standard evaluation system for rice provided by the International Rice Research Institute [41].

### 4.4. Germination and Disease Incidence Scoring 

The number of germinated seedlings in infected plants as well as controls was recorded 4 days after sowing. The disease incidence was recorded by counting the number of infected seedlings in three replications. These data were used to calculate the percent infected seedlings for all the combinations. The height of the plants was also recorded at the same time, along with that of control plants.

(1) $$\mathrm{Disease}\;\mathrm{incidence}=\frac{\mathrm{Number}\;\mathrm{of}\;\mathrm{infected}\;\mathrm{seedlings}}{\mathrm{Total}\;\mathrm{number}\;\mathrm{of}\;\mathrm{seedlings}}\times 100$$

The disease was first noticed 13–14 days after sowing (DAS) in susceptible variety PB1121 and maximum disease was observed at 18 DAS. 

### 4.5. Evaluation and Pathotyping of Fusarium fujikuroi Isolates

Five differential rice genotypes were selected after preliminary screening to characterize the isolates. The disease rating scale (Table 5) was used to classify the disease incidence into different classes of disease reaction patterns and specific pathotype groups. The pathotype groups consisted of isolates showing similar patterns of virulence. Four classes of disease reaction patterns were used for pathotype grouping, i.e., highly resistant (HR), resistant (R), susceptible (S), and highly susceptible (HS). The moderately resistant and moderately susceptible classes were merged into the resistant and susceptible groups, respectively, for pathotype classification. 

### 4.6. Selection of Virulence-Related Genes in Fusarium fujikuroi

Three virulence-related genes were selected to correlate pathotype groups with virulence profiles [29]. Primers were designed through Primer3 Plus software (www.bioinformatics.nl/primer3plus, accessed on 2 May 2022). For qPCR, a product size of 150–250 bp was selected. All primers used in this study are listed in Supplementary Table S10. The primers (forward and reverse) showing the least variation in different parameters such as GC content, melting temperature, and self-complementarity were considered and the specificity was confirmed through NCBI nucleotide blast. The primers were synthesized from Integrated DNA Technologies (IDT, Coralville, IA, USA). Stock primers were stored at −20 °C and they were diluted at the ratio of 1:10 for the experiment. 

### 4.7. Expression Analysis of Virulence-Related Genes in Fusarium fujikuroi

The isolates for RNA extraction were cultivated in potato dextrose broth (PDB) and incubated for 7 days at 25 ± 2 °C. The mycelia of all *Fusarium fujikuroi* isolates were filtered with Whatman No. 1 filter paper fitted into the funnels, and PDB was collected in 250 mL flasks. The mycelium was washed thrice with double-distilled water, immediately collected in liquid nitrogen, and stored at −80 °C until use. RNA was isolated from mycelium according to the Trizol method (Invitrogen, Waltham, MA, USA). Prior to RNA isolation, all laboratory materials, including Eppendorf tubes, spatulas, microcentrifuge tubes, pestles, and mortars were treated with diethyl pyrocarbonate (DEPC) at 0.1 percent to deactivate the RNase enzymes, and materials were autoclaved at 121 °C, 15 psi, for 15 min. Mycelia of *Fusarium fujikuroi* isolates were ground into powders with liquid nitrogen using a pre-cooled mortar and pestle. The fine powder (approximately 100 mg) was transferred into 2 mL Eppendorf tubes containing 1 mL of Trizol reagent and incubated for 5 min at room temperature for the dissociation of the nucleoprotein complex. RNA was extracted manually following the protocol of the Trizol method. The RNA was quantified using a Nano Drop 2000 Spectrophotometer (Thermo Fisher Scientific, Waltham, MA, USA).

Before proceeding to cDNA synthesis, RNA samples were subjected to DNase I treatment, following the manufacturer’s instructions (Thermo Scientific, Waltham, MA, USA), using one µg of total RNA. The treated samples were used for cDNA synthesis with the Verso cDNA synthesis kit, according to the manufacturer’s protocol (Verso cDNA, Thermo Scientific, Waltham, MA, USA). Anchored Oligo dT primers were used for the synthesis of first-strand cDNA. The conditions consisted of one cycle at 42 °C for 30 min and one cycle of inactivation at 95 °C for 2 min. The reverse transcription was performed in a T100 thermal cycler (Bio Rad, Hercules, CA, USA). The cDNA was stored at −20 °C.

The samples for the real-time PCR were set up in 3 replicates along with a non-template control. qPCR plates (Thermo Scientific, Waltham, MA, USA) consisting of 96 wells were used for reactions. The CFX96 real-time system (Bio Rad, Hercules, CA, USA) was used for real-time PCR. DyNAmo flash SYBR (2X) green mix dye (Thermo Scientific, Waltham, MA, USA) was used for fluorescence detection during the amplification. Reactions were set up in dark conditions to protect the fluorescent dye from light sensitivity. The total reaction volume was 10 µL, which consisted 5 µL SYBR, 1 µL forward and reverse primer, 1 µL template cDNA, and 2 µL nuclease-free water. The conditions of the real-time PCR program consisted of initial denaturation at 95 °C for 5 min with 40 cycles of denaturation at 95 °C for 45 s, annealing at 58 °C for 60 s, and default melt curve analysis at 65 °C to 95 °C with 0.5 °C increment/cycle. The target gene expression was quantified using reference gene GAPDH through the 2^−ΔΔCt^ method [42]. The following formula was used to calculate ΔΔCt. One-way ANOVA was used for replicated ΔΔCt values relative gene expression analysis.

ΔΔCt = (C_t target_ − C_t reference_) _sample_ − (C_t target_ − C_t reference_) _control_

### 4.8. Statistical Analysis

The data of disease incidence were subjected to two-way ANOVA (separate analysis for preliminary screening and evaluation of *Fusarium fujikuroi* isolates) using isolate and genotype as independent factors and disease incidence as a dependent factor. The analysis was performed in Microsoft Office Excel v.2019 [43]. The Tukey HSD test was used for multiple comparison of treatments in OPSTAT online analysis statistical software [44]. One-way ANOVA was used for gene expression analysis using replicated ΔΔCt values (Supplementary Table S7). 

## 5. Conclusions

We screened rice genotypes for bakanae disease and identified five distinct rice genotypes differing in resistance. The study characterized a total of 97 isolates of *Fusarium fujikuroi*, collected from different rice-growing states of the country, using a set of rice genotypes differing in their resistance. Based on the disease response in different rice genotypes, a total of 15 pathotypes were identified. Pathotype group 1 was most prevalent, but pathotype group 8 was highly virulent. Pathotypes 11 and 15 were found to originate from Punjab. Six pathotype groups were identified as having correlations between disease severity and virulence-related gene expression data. Further studies on pathotype classification based on morphological and detailed molecular characterization will give a new dimension to the mechanism of variability in *Fusarium fujikuroi* causing bakanae disease in rice. These studies will also be helpful in the development of resistant varieties for disease-affected areas.

## Figures and Tables

**Table 1:** Screening of rice genotypes for bakanae disease of rice caused by *Fusarium fujikuroi*.

Isolate Code	PB 1509	PB 1121	PB-1	Kanak-Jeer	ANP 115-3-3-3-3	C4-63G	K-14	C101A51	PB6	BPT-5204	PS-5	IRG52	DI * Mean
F201	13.79	49.97	1.17	13.56	31.68	0.00	61.15	0.00	10.60	66.81	36.44	4.55	24.14
F209	9.67	83.12	6.03	2.96	5.64	0.00	98.21	0.00	24.74	57.60	57.05	8.35	29.45
F219	25.50	48.11	20.10	6.25	78.39	2.80	35.82	0.00	35.86	50.26	0.00	8.30	25.95
F220	48.18	75.09	14.62	8.16	2.61	0.00	0.00	0.00	36.73	0.00	0.00	7.55	16.08
F224	65.85	58.82	6.89	7.30	23.75	0.00	0.00	0.00	43.42	0.00	0.00	4.80	17.57
F225	28.77	89.72	34.94	9.18	31.50	0.00	23.56	0.00	29.38	22.85	51.99	6.05	27.33
F226	73.94	80.67	26.52	4.98	29.73	0.00	27.06	0.00	34.10	28.63	0.00	0.00	25.47
F228	12.60	53.30	4.61	0.00	4.78	2.75	8.01	0.00	67.64	60.64	31.32	6.56	21.02
F235	27.92	83.67	0.00	7.01	0.00	0.00	4.74	0.00	51.00	17.06	59.00	3.78	21.18
F239	69.41	72.33	16.18	0.00	16.79	0.00	6.58	0.00	38.03	42.16	0.00	4.82	22.19
F250	24.09	76.70	27.13	13.57	0.00	0.00	0.00	0.00	17.90	51.50	0.00	5.40	18.02
F252	74.50	73.24	19.57	0.00	67.87	0.00	63.00	0.00	24.12	66.50	0.00	10.15	33.25
F253	25.22	86.50	31.82	6.52	39.49	0.00	69.87	0.00	65.06	51.32	63.50	6.32	37.13
F255	21.25	91.10	0.00	7.00	0.00	3.13	0.00	0.00	44.10	52.49	48.95	8.78	23.06
F258	46.00	68.35	2.84	6.05	49.35	0.00	95.00	0.00	11.00	37.85	22.50	3.79	28.56
F278	41.38	72.41	12.51	4.25	19.45	0.00	76.65	0.00	28.39	35.12	58.59	0.00	29.06
F344	46.22	59.17	11.99	5.65	6.08	3.90	97.50	0.00	67.69	36.20	0.00	3.50	28.16
F216a	46.33	52.44	23.61	10.17	30.63	0.00	63.56	0.00	66.08	47.11	0.00	7.65	28.96
F225a	46.31	80.89	5.10	1.80	0.00	0.00	4.79	0.00	19.00	0.00	94.90	4.00	21.40
F249a	56.79	77.18	16.00	6.10	0.00	0.00	0.00	0.00	49.50	0.00	52.50	7.50	22.13
DI * Mean	40.18 ^b^	71.64 ^a^	14.08 ^e^	6.02 ^f^	21.89 ^d^	0.63 ^g^	36.77 ^b^	0 ^g^	38.22 ^b^	36.2 ^b^	28.84 ^c^	5.59 ^f^	25.01
DI * pattern	S *	HS *	MS *	MR *	MS *	R *	S *	HR *	S *	S *	S *	MR *	

* DI = disease incidence (%), * S = susceptible, * HS = highly susceptible, * MS = moderately susceptible, * MR = moderately resistant, * R = resistant, and * HR = highly resistant. Alphabetic lowercase letters in DI mean, indicates the significant difference between the genotypes and same letter in two or more genotypes indicates no significant difference.

**Table 2:** Disease incidence (%) in rice genotypes inoculated with *Fusarium fujikuroi* isolates.

S. No.	Isolate No.	PB6	PB-1	C101A51	PB1509	IRG52	DI * Mean Multiple Comparison
1	F201	5.88	17.65	0.00	26.00	7.41	11.39 ^abcd^
2	F204	7.69	6.07	0.00	26.67	7.69	9.62 ^abcd^
3	F205	6.07	7.69	0.00	66.67	8.33	17.75 ^abcd^
4	F206	27.78	33.33	0.00	76.47	12.00	29.92 ^abcd^
5	F206a	7.14	7.69	0.00	41.67	4.35	12.17 ^abcd^
6	F207	5.88	23.53	0.00	43.75	0.00	14.63 ^abcd^
7	F209	6.06	10.00	0.00	77.78	10.00	20.77 ^abcd^
8	F210	5.88	38.89	0.00	46.15	11.54	20.49 ^abcd^
9	F210a	28.57	16.67	0.00	10.53	10.71	13.3 ^abcd^
10	F211	22.22	11.11	0.00	26.32	0.00	11.93 ^abcd^
11	F212a	7.14	16.67	0.00	63.16	7.69	18.93 ^abcd^
12	F213	46.15	57.14	5.88	65.00	12.00	37.24 ^a^
13	F213a	22.22	13.33	0.00	50.00	3.45	17.8 ^abcd^
14	F214	5.88	8.33	0.00	7.14	3.45	4.96 ^bcd^
15	F216	6.09	6.05	0.00	20.00	20.00	10.43 ^abcd^
16	F216a	7.69	26.32	0.00	53.33	12.00	19.87 ^abcd^
17	F217	11.76	10.00	0.00	35.71	0.00	11.5 ^abcd^
18	F217a	21.43	26.67	0.00	35.71	16.00	19.96 ^abcd^
19	F218	6.05	6.09	0.00	7.69	12.00	6.37 ^abcd^
20	F218a	11.11	12.50	0.00	31.25	10.00	12.97 ^abcd^
21	F219	11.11	10.53	0.00	81.25	14.81	23.54 ^abcd^
22	F220	62.50	40.00	0.00	56.25	10.71	33.89 ^abcd^
23	F222	15.38	6.07	0.00	94.12	0.00	23.11 ^abcd^
24	F223	14.29	3.57	0.00	84.62	0.00	20.49 ^abcd^
25	F223a	6.06	7.69	0.00	60.00	12.00	17.15 ^abcd^
26	F224	13.33	7.69	0.00	93.33	7.14	24.3 ^abcd^
27	F225	5.56	5.56	0.00	30.77	7.41	9.86 ^abcd^
28	F225a	5.88	33.33	0.00	22.22	4.00	13.09 ^abcd^
29	F226	50.00	27.27	0.00	93.33	0.00	34.12 ^abc^
30	F228	7.14	16.67	0.00	46.67	7.41	15.58 ^abcd^
31	F228a	20.00	7.69	0.00	30.77	0.00	11.69 ^abcd^
32	F230	20.00	0.00	0.00	36.36	0.00	11.27 ^abcd^
33	F231	6.07	23.08	0.00	42.86	0.00	14.4 ^abcd^
34	F232	12.50	45.45	0.00	35.71	20.69	22.87 ^abcd^
35	F233	40.00	25.00	0.00	92.31	13.33	34.13 ^abc^
36	F234	42.86	33.33	0.00	45.45	7.41	25.81 ^abcd^
37	F235	23.53	25.00	0.00	11.11	3.57	12.64 ^abcd^
38	F237a	5.88	26.67	0.00	36.36	10.71	15.93 ^abcd^
39	F239	66.67	41.18	0.00	66.67	7.41	36.38 ^ab^
40	F240	53.85	41.18	0.00	60.00	10.34	33.07 ^abcd^
41	F241	5.88	5.26	0.00	68.75	3.70	16.72 ^abcd^
42	F242	6.06	5.26	0.00	92.86	6.06	22.05 ^abcd^
43	F242a	16.67	25.00	0.00	92.31	15.38	29.87 ^abcd^
44	F244	29.41	11.76	5.26	62.50	10.71	23.93 ^abcd^
45	F245a	9.09	11.76	0.00	33.33	0.00	10.84 ^abcd^
46	F246	27.27	35.71	0.00	46.67	18.18	25.57 ^abcd^
47	F247	31.25	41.67	0.00	27.27	10.00	22.04 ^abcd^
48	F249	23.53	33.33	0.00	36.84	3.57	19.46 ^abcd^
49	F249a	23.08	5.26	0.00	22.22	7.41	11.59 ^abcd^
50	F250	23.08	15.38	5.26	44.44	7.14	19.06 ^abcd^
51	F252	16.67	5.58	0.00	7.77	12.50	8.5 ^abcd^
52	F253	21.43	15.38	0.00	33.33	3.57	14.74 ^abcd^
53	F254	35.71	21.43	0.00	21.43	17.86	19.29 ^abcd^
54	F255	14.29	6.06	0.00	22.22	11.11	10.74 ^abcd^
55	F255a	6.07	5.26	0.00	5.26	0.00	3.32 ^cd^
56	F256	23.53	7.69	0.00	93.33	7.41	26.39 ^abcd^
57	F258	9.09	5.88	0.00	50.00	3.33	13.66 ^abcd^
58	F259	21.43	12.50	0.00	40.00	7.69	16.32 ^abcd^
59	F261	31.25	33.33	0.00	41.67	3.57	21.96 ^abcd^
60	F263	25.00	7.69	0.00	12.50	25.00	14.04 ^abcd^
61	F267	6.07	7.14	0.00	54.55	11.54	15.86 ^abcd^
62	F268	23.53	12.50	0.00	31.25	7.41	14.94 ^abcd^
63	F272	28.57	5.88	0.00	33.33	4.00	14.36 ^abcd^
64	F274	8.33	23.08	6.07	21.43	18.52	15.48 ^abcd^
65	F277	28.57	21.43	6.07	21.43	20.83	19.67 ^abcd^
66	F278	7.69	10.00	0.00	33.33	0.00	10.21 ^abcd^
67	F278a	20.00	6.25	0.00	33.33	3.84	12.68 ^abcd^
68	F279	8.33	4.00	0.00	33.33	3.85	9.9 ^abcd^
69	F280	8.33	5.26	0.00	8.33	20.69	8.52 ^abcd^
70	F282	25.00	6.25	0.00	35.71	25.92	18.58 ^abcd^
71	F284	7.14	3.33	0.00	16.67	22.22	9.87 ^abcd^
72	F285	35.29	10.00	0.00	26.32	0.00	14.32 ^abcd^
73	F287	18.18	6.06	0.00	9.09	3.57	7.38 ^abcd^
74	F288	14.29	7.69	0.00	21.43	4.00	9.48 ^abcd^
75	F289	18.75	33.33	0.00	9.09	24.14	17.06 ^abcd^
76	F291	46.67	6.07	0.00	8.33	21.43	16.5 ^abcd^
77	F294	0.00	0.00	0.00	33.33	3.85	7.44 ^abcd^
78	F295	25.00	3.33	0.00	7.69	7.41	8.69 ^abcd^
79	F297	0.00	0.00	0.00	15.38	6.90	4.46 ^cd^
80	F298	7.69	16.67	0.00	11.76	0.00	7.22 ^abcd^
81	F300	18.18	7.69	0.00	27.27	3.70	11.37 ^abcd^
82	F309	26.66	5.88	0.00	33.33	3.44	13.86 ^abcd^
83	F339	25.00	23.53	0.00	10.00	17.86	15.28 ^abcd^
84	F340	7.14	9.09	0.00	20.00	29.63	13.17 ^abcd^
85	F341	18.75	9.09	0.00	10.00	11.11	9.79 ^abcd^
86	F342	28.57	25.00	0.00	10.00	14.81	15.68 ^abcd^
87	F343	26.32	8.33	5.88	40.00	7.41	17.59 ^abcd^
88	F344	0.00	0.00	0.00	7.69	3.57	2.25 ^d^
89	Bundi-1	18.75	12.50	0.00	50.00	0.00	16.25 ^abcd^
90	Bundi-2	20.00	25.00	0.00	38.46	10.71	18.84 ^abcd^
91	Bundi-3	45.45	45.45	0.00	10.00	3.57	20.9 ^abcd^
92	Bundi-4	42.86	18.18	0.00	11.76	7.41	16.04 ^abcd^
93	Bundi-5	11.76	33.33	0.00	55.56	7.14	21.56 ^abcd^
94	NIB *	22.22	5.88	0.00	75.00	0.00	20.62 ^abcd^
95	F1121	40.00	18.18	0.00	21.43	0.00	15.92 ^abcd^
96	F1728	23.53	10.00	0.00	78.57	0.00	22.42 ^abcd^
97	Bundi	29.41	13.33	0.00	45.45	0.00	17.64 ^abcd^
DI * Mean		19.52	16.01	0.35	39.13	8.35	16.67

* NIB = new isolate Bareilly, * DI = disease incidence (%). Alphabetic lowercase letters in DI mean, indicates the significant difference between the isolates and same letter in two or more isolates indicates no significant difference.

**Table 3:** Pathotype classification of *Fusarium fujikuroi* isolates based on pattern of disease reaction in rice genotypes differing in their resistance.

Pathotypes	Rice Genotypes					Isolates	Total Isolates
	PB6	PB1	C101A51	PB1509	IRG52		
1	S	S	HR	S	S	F210a, F217a, F218a, F228, F232, F234, F246, F247, F254, F259, F263, F268, F289, F339, F340, F341, F342, Bundi-2, Bundi-4	19
2	S	S	HR	S	HR	F211, F217, F226, F228a, F230, F245a, F278, F285, F298, Bundi, Bundi-1, F1121, F1728	13
3	S	S	HR	S	R	F206a, F213a, F235, F249, F253, F261, F288, F300, Bundi-3	9
4	S	S	HR	HS	S	F206, F212a, F216a, F219, F224, F233, F242a, F256, Bundi-5	9
5	S	R	HR	S	S	F204, F249a, F252, F255, F255a, F280, F282, F284, F291, F295	10
6	R	S	HR	HS	S	F205, F209, F223a, F267	4
7	S	R	HR	S	R	F258, F272, F278a, F279, F287, F309	6
8	S	S	R	S	S	F213, F244, F250, F274, F277, F343	6
9	R	S	HR	S	S	F201, F210, F237a	3
10	S	R	HR	HS	HR	F222, F223, NIB	3
11	R	R	HR	S	S	F216, F218, F225, F225a	4
12	HS	S	HR	HS	S	F220, F239, F240	3
13	HR	HR	HR	S	R	F294, F297, F344	3
14	R	S	HR	S	HR	F207, F214, F231	3
15	R	R	HR	HS	R	F241, F242	2
							97

where S = susceptible, HS = highly susceptible, R = resistant, and HR = highly resistant.

**Table 4:** Geographical distribution of *Fusarium fujikuroi* pathotypes.

Geographical Distribution	Pathotypes															Total Isolates
	1	2	3	4	5	6	7	8	9	10	11	12	13	14	15	
Punjab	9	7	6	6	4	3	5	4	2	2	4	2	2	1	2	59
Haryana	3	0	2	1	3	0	0	2	0	0	0	0	0	1	0	12
Uttar Pradesh	2	1	0	1	1	1	1	0	1	1	0	0	1	0	0	10
Uttarakhand	3	1	0	0	2	0	0	0	0	0	0	1	0	1	0	8
Rajasthan	2	2	1	1	0	0	0	0	0	0	0	0	0	0	0	6
Delhi	0	2	0	0	0	0	0	0	0	0	0	0	0	0	0	2
Total isolates	19	13	9	9	10	4	6	6	3	3	4	3	3	3	2	97

**Table 5:** Bakanae disease evaluation method according to the standard evaluation system of the International Rice Research Institute [41].

Disease Rating Scale	Percent Infected Seedlings	Disease Reaction
0	0%	Highly resistant (HR)
1	≤1%	Resistant (R)
3	1–6%	Moderately resistant (MR)
5	6–25%	Moderately susceptible (MS)
7	25–50%	Susceptible (S)
9	50–100%	Highly susceptible (HS)

## Data Availability

All data generated during this study are available in the published manuscript and its Supplementary Information File.

## Supplementary Materials

The following supporting information can be downloaded at: https://www.mdpi.com/article/10.3390/plants12061303/s1, Figure S1: Healthy rice genotypes (uninoculated control plants) used in this study; Table S1: ANOVA for preliminary screening of rice genotypes inoculated with *Fusarium fujikuroi* isolates; Table S2: Tukey’s HSD Test for comparison of rice genotypes in preliminary screening; Table S3: ANOVA for elongation of the rice genotypes due to infection by *Fusarium fujikuroi*; Table S4: Disease incidence pattern in rice genotypes differing for their resistance; Table S5: ANOVA for evaluation of *Fusarium fujikuroi* isolates; Table S6: Tukey’s HSD Test for comparison of *Fusarium fujikuroi* isolates; Table S7: One way ANOVA for virulence-related gene expression analysis in *Fusarium fujikuroi*; Table S8: *Fusarium fujikuroi* isolates, their place, year and source of collection [6,27]; Table S9: Rice genotypes used in this study; Table S10: PCR and qPCR primers used in this study [30].
