# Supplementary material for: Identification and Characterization of Fusarium fujikuroi Pathotypes Responsible for an Emerging Bakanae Disease of Rice in India
Source: Plants (Basel). 2023 Mar 14;12(6):1303. doi: 10.3390/plants12061303 (PMC10059007; doi:10.3390/plants12061303)
Supplement: Supplementary file 1 [file plants-12-01303-s001.zip › plants-1897641-supplementary.pdf]

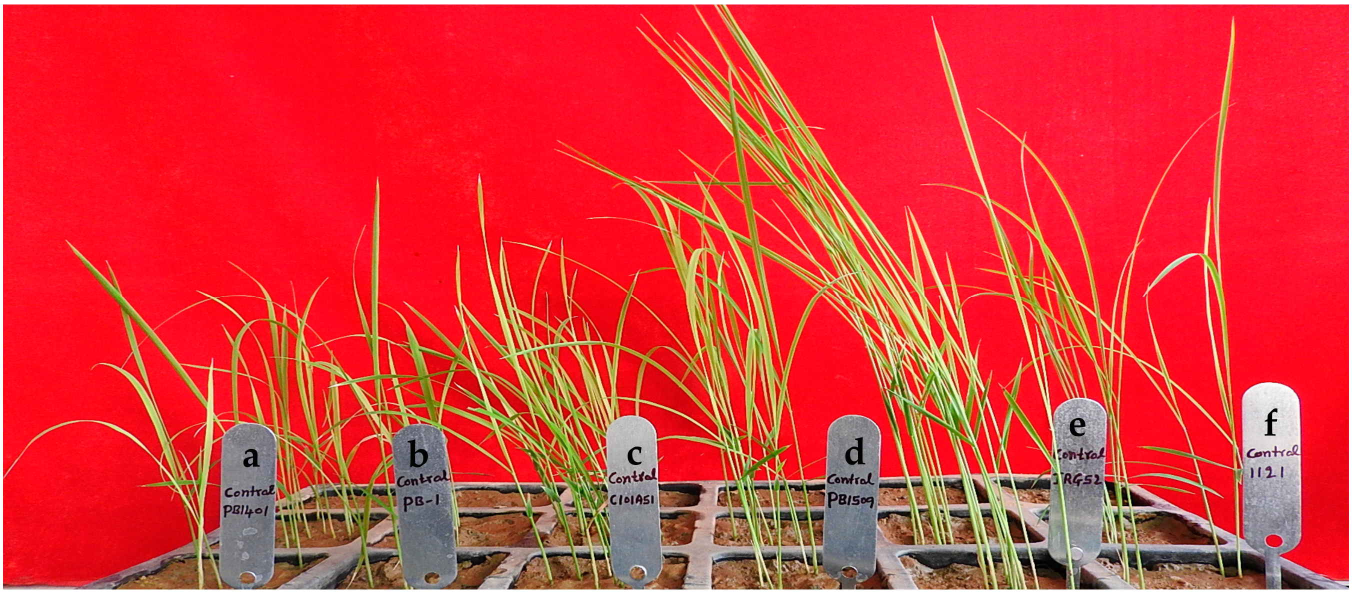

Supplementary Figure. S1 Healthy rice genotypes (uninoculated control plants) used in this study.
a: PB6 (PB1401), **b:** PB1, **c:** C101A51, **d:** PB1509, **e:** IRG52 and **f:** PB1121

Supplementary Table S1 ANOVA for preliminary screening of rice genotypes inoculated with *Fusarium fujikuroi* isolates

ANOVA

Source of Variation	*SS*	*df*	*MS*	*F*	*P-value*	*F crit*
Genotype	197753.7	11	17977.61	1096.762*	2.9E-198	1.828695
Isolates	13052.72	19	686.985	41.91095*	3.49E-65	1.630056
Interaction	155793.2	209	745.4222	45.47603*	4E-129	1.245555
Within	3933.97	240	16.39154			
Total	370533.7	479				

*Significant p=0.05 (5% level of significance)

**Factors**	**C.D.**	**SE(d)**	**SE(m)**
Isolates	2.309	1.171	0.828
Genotypes	1.788	0.907	0.641
Interaction	7.997	4.057	2.869

Supplementary Table S2 Tukey's HSD Test for comparison of rice genotypes in preliminary screening

Treat No	Treat Name	Treat Mean	Least Sign.Diff
1	PB1509	40.18^{b}	4.226
2	PB1121	71.64^{a}	4.226
3	PB1	14.08^{e}	4.226
4	Kanak-Jeer	6.02^{f}	4.226
5	ANP 115-3-3-3-3	21.89^{d}	4.226
6	C4-63G	0.63^{g}	4.226
7	K-14	36.77^{b}	4.226
8	C101A51	0^{g}	4.226
9	PB6	38.22^{b}	4.226
10	BPT-5204	36.2^{b}	4.226
11	PS-5	28.84^{c}	4.226
12	IRG52	5.59^{f}	4.226

Alphabetic lowercase letters in Treatment mean, indicates the significant difference between the genotypes and same letter in two or more genotypes indicates no significant difference.

Supplementary Table S3 ANOVA for elongation of the rice genotypes due to infection by *Fusarium fujikuroi*

ANOVA

Source of Variation	*SS*	*df*	*MS*	*F*	*P-value*	*F crit*
Genotypes	46337.6	4	11584.4	663.7226*	0	2.375597
Isolates	2709.401	96	28.22292	1.617019*	0.000181	1.255279
Interaction	8086.737	384	21.05921	1.206577*	0.006366	1.132524
Within	42325.17	2425	17.45368			
Total	99458.9	2909				

*Significant p=0.05 (5% level of significance)

Supplementary Table S4 Disease incidence pattern in rice genotypes differing for their resistance

Ff Isolate No	PB6	PB1	C101A51	PB1509	IRG52
F201	R	S	HR	S	S
F204	S	R	HR	S	S
F205	R	S	HR	HS	S
F206	S	S	HR	HS	S
F206a	S	S	HR	S	R
F207	R	S	HR	S	HR
F209	R	S	HR	HS	S
F210	R	S	HR	S	S
F210a	S	S	HR	S	S
F211	S	S	HR	S	HR
F212a	S	S	HR	HS	S
F213	S	S	R	HS	S
F213a	S	S	HR	S	R

F214	R	S	HR	S	R
F216	R	R	HR	S	S
F216a	S	S	HR	HS	S
F217	S	S	HR	S	HR
F217a	S	S	HR	S	S
F218	R	R	HR	S	S
F218a	S	S	HR	S	S
F219	S	S	HR	HS	S
F220	HS	S	HR	HS	S
F222	S	R	HR	HS	HR
F223	S	R	HR	HS	HR
F223a	R	S	HR	HS	S
F224	S	S	HR	HS	S
F225	R	R	HR	S	S
F225a	R	S	HR	S	R
F226	S	S	HR	HS	HR
F228	S	S	HR	S	S
F228a	S	S	HR	S	HR
F230	S	S	HR	S	HR
F231	R	S	HR	S	HR
F232	S	S	HR	S	S
F233	S	S	HR	HS	S
F234	S	S	HR	S	S
F235	S	S	HR	S	R
F237a	R	S	HR	S	S
F239	HS	S	HR	HS	S
F240	HS	S	HR	HS	S
F241	R	R	HR	HS	R
F242	R	R	HR	HS	R
F242a	S	S	HR	HS	S
F244	S	S	R	HS	S
F245a	S	S	HR	S	HR
F246	S	S	HR	S	S
F247	S	S	HR	S	S
F249	S	S	HR	S	R
F249a	S	R	HR	S	S
F250	S	S	R	S	S
F252	S	R	HR	S	S
F253	S	S	HR	S	R
F254	S	S	HR	S	S
F255	S	R	HR	S	S
F255a	R	R	HR	R	HR
F256	S	S	HR	HS	S
F258	S	R	HR	S	R
F259	S	S	HR	S	S
F261	S	S	HR	S	R
F263	S	S	HR	S	S
F267	R	S	HR	HS	S
F268	S	S	HR	S	S

F272	S	R	HR	S	R
F274	S	S	R	S	S
F277	S	S	R	S	S
F278	S	S	HR	S	HR
F278a	S	R	HR	S	R
F279	S	R	HR	S	R
F280	S	R	HR	S	S
F282	S	R	HR	S	S
F284	S	R	HR	S	S
F285	S	S	HR	S	HR
F287	S	R	HR	S	R
F288	S	S	HR	S	R
F289	S	S	HR	S	S
F291	S	R	HR	S	S
F294	HR	HR	HR	S	R
F295	S	R	HR	S	S
F297	HR	HR	HR	S	R
F298	S	S	HR	S	HR
F300	S	S	HR	S	R
F309	S	R	HR	S	R
F339	S	S	HR	S	S
F340	S	S	HR	S	S
F341	S	S	HR	S	S
F342	S	S	HR	S	S
F343	S	S	R	S	S
F344	HR	HR	HR	S	R
Bundi-1	S	S	HR	S	HR
Bundi-2	S	S	HR	S	S
Bundi-3	S	S	HR	S	R
Bundi-4	S	S	HR	S	S
Bundi-5	S	S	HR	HS	S
NIB	S	R	HR	HS	HR
F1121	S	S	HR	S	HR
F1728	S	S	HR	HS	HR
Bundi	S	S	HR	S	HR

Where, S= Susceptible, HS= Highly susceptible, R= Resistant and HR= Highly resistant

Supplementary Table S5 ANOVA for evaluation of *Fusarium fujikuroi* isolates

ANOVA

Source of Variation	*SS*	*df*	*MS*	*F*	*P-value*	*F crit*
Genotypes	154259.3	4	38564.83	290.21*	3.6E-127	2.390321
Isolates	54139.43	96	563.9524	4.243883*	2.86E-26	1.281112
Interaction	163644.2	384	426.1567	3.206936*	1.82E-33	1.171427
Within	64449.68	485	132.8859			
Total	436492.6	969				

*Significant p=0.05 (5% level of significance)

Factors	C.D.	SE(d)	SE(m)
Isolates	10.251	5.215	3.687
Genotypes	2.364	1.203	0.850
Interaction	22.922	11.661	8.245

Supplementary Table S6 Tukey's HSD Test for comparison of *Fusarium fujikuroi* isolates

Treat No	Treat Name	Treat Mean
1	201	11.39[abcd]
2	204	9.62[abcd]
3	205	17.75[abcd]
4	206	29.92[abcd]
5	206a	12.17[abcd]
6	207	14.63[abcd]
7	209	20.77[abcd]
8	210	20.49[abcd]
9	210a	13.3[abcd]
10	211	11.93[abcd]
11	212a	18.93[abcd]
12	213	37.24[a]
13	213a	17.8[abcd]
14	214	4.96[bcd]
15	216	10.43[abcd]
16	216a	19.87[abcd]
17	217	11.5[abcd]
18	217a	19.96[abcd]
19	218	6.37[abcd]
20	218a	12.97[abcd]
21	219	23.54[abcd]
22	220	33.89[abc]
23	222	23.11[abcd]
24	223	20.49[abcd]
25	223a	17.15[abcd]
26	224	24.3[abcd]
27	225	9.86[abcd]
28	225a	13.09[abcd]
29	226	34.12[abc]
30	228	15.58[abcd]
31	228a	11.69[abcd]
32	230	11.27[abcd]
33	231	14.4[abcd]
34	232	22.87[abcd]
35	233	34.13[abc]

36	234	25.81abcd
37	235	12.64abcd
38	237a	15.93abcd
39	239	36.38ab
40	240	33.07abcd
41	241	16.72abcd
42	242	22.05abcd
43	242a	29.87abcd
44	244	23.93abcd
45	245a	10.84abcd
46	246	25.57abcd
47	247	22.04abcd
48	249	19.46abcd
49	249a	11.59abcd
50	250	19.06abcd
51	252	8.5abcd
52	253	14.74abcd
53	254	19.29abcd
54	255	10.74abcd
55	255a	3.32cd
56	256	26.39abcd
57	258	13.66abcd
58	259	16.32abcd
59	261	21.96abcd
60	263	14.04abcd
61	267	15.86abcd
62	268	14.94abcd
63	272	14.36abcd
64	274	15.48abcd
65	277	19.67abcd
66	278	10.21abcd
67	278a	12.68abcd
68	279	9.9abcd
69	280	8.52abcd
70	282	18.58abcd
71	284	9.87abcd
72	285	14.32abcd
73	287	7.38abcd
74	288	9.48abcd
75	289	17.06abcd
76	291	16.5abcd
77	294	7.44abcd
78	295	8.69abcd
79	297	4.46cd
80	298	7.22abcd
81	300	11.37abcd

82	309	13.86[abcd]
83	339	15.28[abcd]
84	340	13.17[abcd]
85	341	9.79[abcd]
86	342	15.68[abcd]
87	343	17.59[abcd]
88	344	2.25[d]
89	Bundi-1	16.25[abcd]
90	Bundi-2	18.84[abcd]
91	Bundi-3	20.9[abcd]
92	Bundi-4	16.04[abcd]
93	Bundi-5	21.56[abcd]
94	NIB	20.62[abcd]
95	1121	15.92[abcd]
96	1728	22.42[abcd]
97	Bundi	17.64[abcd]

Alphabetic lowercase letters in Treatment mean, indicates the significant difference between the isolates and same letter in two or more isolates indicates no significant difference.

Least Significant Difference (LSD): 31.51

Supplementary Table S7 One way ANOVA for virulence-related gene expression analysis in *Fusarium fujikuroi*

ANOVA: *FFAC*

Source of Variation	*SS*	*df*	*MS*	*F*	*P-value*	*F crit*
Between Groups	307.8079	19	16.20042	3.461719*	0.000465	1.8528918
Within Groups	187.1951	40	4.679877			
Total	495.003	59				

ANOVA: *FFEX*

Source of Variation	*SS*	*df*	*MS*	*F*	*P-value*	*F crit*
Between Groups	488.1971	19	25.69458	6.556204*	3.13E-07	1.852892
Within Groups	156.765	40	3.919125			
Total	644.9621	59				

ANOVA: *FFPD*

Source of Variation	*SS*	*df*	*MS*	*F*	*P-value*	*F crit*
Between Groups	209.4105	19	11.02161	4.402568*	3.94E-05	1.852892
Within Groups	100.138	40	2.503449			
Total	309.5485	59				

*Significant p=0.05 (5% level of significance)

**Virulence-related genes in *Fusarium fujikuroi***	**ErMs**	**SE**	**SEd**	**CD@5%**
**FFAC**	4.6798765	1.2489831	1.7663289	3.6969688

**FFEX**	3.9191254	1.1429677	1.6164004	3.5576733
**FFPD**	2.5034494	0.9135005	1.2918848	2.8434192

Supplementary Table S8 *Fusarium fujikuroi* isolates, their place, year and source of collection [6,27]

SL. N0.	Isolate No	Place of collection	Year of collection	Rice variety
1	F201	Punjab (Fatehgarh)	2011	Pusa Basmati 6
2	F204	Punjab (Fatehgarh)	2011	Pusa Basmati 6
3	F205	Punjab	2011	Pusa Basmati 1121
4	F206	Punjab (Tarn Taran)	2011	Pusa Basmati 1121
5	F206a	Punjab (Tarn Taran)	2011	Pusa Basmati 1121
6	F207	Uttarakhand	2012	Pakistani Basmati
7	F209	Uttar Pradesh (Bulandshehar)	2012	Pusa Basmati 1121
8	F210	Uttar Pradesh (Bulandshehar)	2012	Pusa Basmati 1121
9	F210a	Uttar Pradesh (Bulandshehar)	2012	Pusa Basmati 1121
10	F211	Uttar Pradesh (Bulandshehar)	2012	Pusa Basmati 1121
11	F212a	Punjab	2011	Pusa Basmati 1121
12	F213	Punjab	2011	Pusa Basmati 1121
13	F213a	Punjab	2011	Pusa Basmati 1121
14	F214	Haryana (Hisar)	2011	Pusa Basmati 1121

15	F216	Punjab (Kapurthala)	2011	Pusa Basmati 1121
16	F216a	Punjab (Jalandhar)	2011	Pusa Basmati 1121
17	F217	Punjab (Jalandhar)	2011	Pusa Basmati 1121
18	F217a	Punjab	2011	Pusa Basmati 1121
19	F218	Punjab	2011	Pusa Basmati 1121
20	F218a	Punjab (Rajpura)	2011	Pusa Basmati 1121
21	F219	Punjab (Rajpura)	2011	Pusa Basmati 1121
22	F220	Punjab (Sangrur)	2011	Pusa Basmati 1121
23	F222	Punjab	2011	Pusa Basmati 1121
24	F223	Punjab	2011	Pusa Basmati 1121
25	F223a	Punjab	2011	Pusa Basmati 1121
26	F224	Punjab (Sangrur)	2011	Pusa Basmati 1121
27	F225	Punjab (Sangrur)	2011	Pusa Basmati 1121
28	F225a	Punjab	2011	Pusa Basmati 1121
29	F226	Punjab (Kapurthala)	2011	Pusa Basmati 1121
30	F228	Punjab (Tarn Taran)	2011	Pusa Basmati 1121
31	F228a	Punjab	2011	Pusa Basmati 1121
32	F230	Punjab	2011	Pusa Basmati 1121
33	F231	Punjab (Fatehgarh)	2011	Pusa Basmati 1121
34	F232	Uttar Pradesh (Bulandshehar)	2012	Pusa Basmati 1509

35	F233	Uttar Pradesh	2012	Pusa Basmati 1509
36	F234	Punjab (Sangrur)	2011	Pusa Basmati 1121
37	F235	Punjab (Sangrur)	2011	Pusa Basmati 1121
38	F237a	Punjab (Sangrur)	2011	Pusa Basmati 1121
39	F239	Punjab (Abohar)	2011	Pusa Basmati 1121
40	F240	Uttarkhand	2012	Sarbati
41	F241	Punjab (Firozpur)	2011	Pusa Basmati 1121
42	F242	Punjab (Firozpur)	2011	Pusa Basmati 1121
43	F242a	Punjab	2011	Pusa Basmati 1121
44	F244	Punjab	2011	Pusa Basmati 1121
45	F245a	Punjab	2011	Pusa Basmati 1121
46	F246	Punjab	2011	Pusa Basmati 1121
47	F247	Punjab (Jalandhar)	2011	Pusa Basmati 1121
48	F249	Haryana (Sirsa)	2011	Pusa Basmati 1121
49	F249a	Haryana	2011	Pusa Basmati 1121
50	F250	Haryana (Sirsa)	2011	Pusa Basmati 1121
51	F252	Haryana (Karnal)	2011	Pusa Basmati 1121
52	F253	Haryana (Sirsa)	2011	Pusa Basmati 1121
53	F254	Haryana	2011	Pusa Basmati 1121
54	F255	Uttarkhand	2012	Sarbati

55	F255a	Uttarakhand (Ahmedpur)	2012	Sarbati
56	F256	Haryana (Kurukshetra)	2011	Pusa Basmati 1121
57	F258	Punjab (Taran Taran)	2011	Pusa Basmati 1121
58	F259	Punjab (Tarn Taran)	2011	Pusa Basmati 1121
59	F261	Punjab	2011	Pusa Basmati 1121
60	F263	Punjab	2011	Pusa Basmati 1121
61	F267	Punjab (Malhoaut)	2011	Pusa Basmati 1121
62	F268	Punjab (Abohar)	2011	Pusa Basmati 1121
63	F272	Punjab (Muktsar)	2011	Pusa Basmati 1121
64	F274	Punjab (Sangrur)	2011	Pusa Basmati 1121
65	F277	Haryana	2011	Pusa Basmati 1121
66	F278	Punjab (Taran Taran)	2011	Pusa Basmati 1121
67	F278a	Punjab (Taran Taran)	2011	Pusa Basmati 1121
68	F279	Punjab (Kapurthala)	2011	Pusa Basmati 1121
69	F280	Punjab	2011	Pusa Basmati 1121
70	F282	Haryana (Hisar)	2011	Pusa Basmati 1121
71	F284	Uttar Pradesh (Bulandshehar)	2012	Pusa Basmati 1121
72	F285	Punjab	2011	Pusa Basmati 1121
73	F287	Punjab	2011	Pusa Basmati 1121

74	F288	Punjab	2011	Pusa Basmati 1121
75	F289	Haryana	2011	Pusa Basmati 1121
76	F291	Punjab	2011	Pusa Basmati 1121
77	F294	Uttar Pradesh	2012	Pusa Basmati 1121
78	F295	Punjab (Bhudan)	2011	Pusa Basmati 1121
79	F297	Punjab (Kapurthala)	2011	Pusa Basmati 1121
80	F298	Uttarkhand (Ahmedpur)	2012	Sarbati
81	F300	Punjab (Taran Taran)	2011	Pusa Basmati 1121
82	F309	Uttar Pradesh (Aligarh)	2011	Pusa Basmati 6
83	F339	Uttarkhand	2012	Sarbati
84	F340	Haryana	2011	Pusa Basmati 1121
85	F341	Uttarakhand (Ahmedpur)	2012	Pakistani Basmati
86	F342	Uttarkhand	2012	Sarbati
87	F343	Punjab (Sangrur)	2011	Pusa Basmati 1121
88	F344	Punjab	2011	Pusa Basmati 1121
89	Bundi-1	Rajasthan (Bundi)	2018	Pusa Basmati 1509
90	Bundi-2	Rajasthan (Bundi)	2018	Pusa Basmati 1509
91	Bundi-3	Rajasthan (Bundi)	2018	Pusa Basmati 1509
92	Bundi-4	Rajasthan (Bundi)	2018	Pusa Basmati 1509
93	Bundi-5	Rajasthan (Bundi)	2018	Pusa Basmati 1509

94	NIB*	Uttar Pradesh (Bareilly)	2018	Pusa Basmati 1509
95	F1121	New Delhi (IARI)	2020	Pusa Basmati 1121
96	F1728	New Delhi (IARI)	2020	Pusa Basmati 1728
97	Bundi	Rajasthan (Bundi)	2020	Pusa Basmati 1509

*New Isolate Bareilly

Supplementary Table S9 Rice genotypes used in this study

Sl.No.	Name of the genotype
1	Kanak-Jeer
2	ANP 115-3-3-3-3
3	C4-63G
4	BPT5204
5	C101A51
6	IRG52
7	K-14
8	PB1
9	PB1121
10	PB6
11	PB1509
12	PS-5

Supplementary Table S10 PCR and qPCR primers used in this study [30]

S. No.	Primer Name	Primer sequence
1	FFEX_F	AGTTGTTGGCACCTCGTCTC
	FFEX_R	CTTGCTTGGAACCTGGACTC
2	FFAC_F	TGAAACTTCAGTCGCACCAG
	FFAC_R	GCAGCAGAAGGGATCTTGAG
3	FFPD_F	GGCTGCAGATTCTTTTCTGG
	FFPD_R	ACCGTCTCAACAACTGCACA
4	FFEX_RT_F	CCACGAGAATAACGACAACG
	FFEX_RT_R	TTCTTGCTTGGAACCTGGAC
5	FFAC_RT_F	TGCAGTCCAACAGGTTAAGG
	FFAC_RT_R	ATGCTGACGTATTGCTGGTG
6	FFPD_RT_F	AAGGATAGGCTTGGGTTTGG
